# Total thrombus formation system exploration of primary hemostasis in cirrhotic patients

**DOI:** 10.1016/j.rpth.2026.103370

**Published:** 2026-01-29

**Authors:** Johan Abdoul, Norman Luc, Bérangère S. Joly, Antoine Jehl, Adeline Blandinières, Frédéric Adam, Léa Duhaut, Rania Aljhni, Lamiae Grimaldi, Peter J. Lenting, Anirban Sen Gupta, Cécile V. Denis, Stéphanie Roullet

**Affiliations:** 1 Université Paris-Saclay, INSERM, Hémostase Inflammation Thrombose HITh U1176, Le Kremlin-Bicêtre, France; 2 Department of Biomedical Engineering, Case Western Reserve University, Cleveland, Ohio, USA; 3 Service d’Hématologie Biologique Hôpital Lariboisière, AP-HP Nord, Paris, France; 4 INSERM UMRS1138, Centre de Recherche des Cordeliers, Université Paris Cité, Sorbonne Université, Paris, France; 5 Service d’Hématologie Biologique, Hôpital Bicêtre, AP-HP, Le Kremlin-Bicêtre, France; 6 Centre hépato-biliaire Henri Bismuth, Hôpital Paul Brousse, AP-HP, Villejuif, France; 7 Service de Biochimie et Oncogénétique, Hôpital Paul Brousse, AP-HP, Villejuif, France; 8 Clinical Research Unit, Université Paris Saclay, Hôpital Bicêtre, AP-HP, Le Kremlin-Bicêtre, France; 9 CHRU de Nancy, Nancy, France; 10 Service d’Anesthésie-Réanimation, Hôpital Paul Brousse, AP-HP, Villejuif, France

**Keywords:** liver cirrhosis, primary hemostasis, platelets, platelet-mimicking nanoparticles, total thrombus formation system, von Willebrand factor

## Abstract

**Background:**

Primary hemostasis is impaired in cirrhosis, characterized by thrombocytopenia, platelet dysfunction, elevated von Willebrand Factor (VWF) levels, and reduced a disintegrin-like metalloproteinase with thrombospondin type-1 motifs 13 (ADAMTS13) activity. Current clinical hemostasis tests assess biological parameters individually or use static clot formation models.

**Objectives:**

We aimed to study thrombus formation in patients with cirrhosis in whole blood under flow conditions with the Total Thrombus Formation Analysis System (T-TAS 01). We also investigated whether synthetic platelet-mimicking nanoparticles (PMNPs) could improve primary hemostatic function in cirrhotic samples.

**Methods:**

This observational study included 60 participants (30 patients with cirrhosis and 30 controls). All patients underwent blood cell counts, VWF profiling (antigen, activity, propeptide levels, and multimer analysis), ADAMTS13 activity measurement, and T-TAS 01 analysis using collagen-coated PL chips. In cirrhotic samples, T-TAS 01 assays were also performed after adding PMNPs to evaluate their hemostatic efficacy.

**Results:**

Patients with cirrhosis had significantly lower platelet counts (97 vs 218 G/L, *P* < .0001), higher VWF antigen levels (310% vs 119%, *P* < .0001), and reduced ADAMTS13 activity (77% vs 100%, *P* < .01) compared with controls. T-TAS 01 showed prolonged occlusion start time (5:08 vs 2:21 min:sec, *P* < .0001) and occlusion time (10:00 vs 5:00, *P* < .0001), with reduced AUC at 10 minutes (96 vs 382, *P* < .0001). These impairments were more pronounced in patients with higher Child-Pugh scores. PMNPs showed only partial effects on T-TAS 01 parameters.

**Conclusion:**

T-TAS 01 effectively detects hemostatic impairment in patients with cirrhosis, which apparently is not compensated by the presence of elevated VWF levels. This approach may therefore serve as a valuable tool for assessing clinical and biological monitoring in patients with cirrhosis.

## Essentials

• Patients with cirrhosis suffer from impaired primary hemostasis and thrombocytopenia.
• Current clinical tests do not provide a full overview of their hemostatic function.
• T-TAS01 is an innovative method to assess primary hemostasis of these patients.
• Synthetic platelet-mimicking nanoparticles failed to improve hemostasis as measured by T-TAS01.

## Graphical abstract

**Figure:**
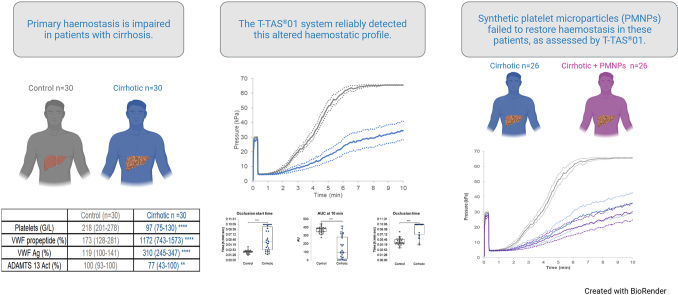

## 1 Introduction

Cirrhosis is associated with an alteration of the hemostatic balance [1]. In the context of cirrhosis and portal hypertension, primary hemostatic abnormalities often present as moderate thrombocytopenia. This results from a combination of mechanisms, including reduced platelet production due to decreased thrombopoietin synthesis and in some cases bone marrow suppression (alcohol and viral infection), platelet splenic sequestration, and in selected settings immune-mediated platelet destruction [2]. In addition, conflicting reports have been published regarding platelet function, which can be altered or enhanced [[3], [4], [5], [6], [7], [8]] during cirrhosis. In parallel with these abnormalities, an increase in von Willebrand factor (VWF) antigen levels have been described and appears to preserve the capacity for platelet aggregation, despite a reduction in its intrinsic functional activity. This increase in VWF antigen can be explained by increased synthesis due to a larger endothelial surface area in the presence of collateral circulation, as well as repeated endothelial injury caused by endotoxemia during infections [1,[9], [10], [11], [12]]. While reduced a disintegrin and metalloprotease with thrombospondin type-1 repeats, member 13 (ADAMTS13) levels, this does not necessarily result in an accumulation of high molecular weight multimers. In liver disease, a reduction of these multimers has been reported, likely due to ADAMTS13-independent proteolysis, involving the central effector of fibrinolysis plasmin, or their consumption within platelet-rich microthrombi [[13], [14], [15]]. Routine investigation of abnormalities in primary hemostasis is based almost exclusively on platelet counts, as well as plasma VWF and ADAMTS13 antigenic and/or activity assays. Functional platelet tests (platelet closure time on the Platelet Function Analyzer, impedance aggregometry, and light transmission aggregation) are not routinely performed, not only because of the significant handling they require, but also because of the additional difficulty posed by thrombocytopenia. These laboratory assays analyze separately the different aspects of platelet functions, without assessing vessel walls or endothelial cells [3]. Importantly, all of these assays are conducted under static conditions and fail to replicate the dynamic environment of the circulation [16,17]. By contrast, a flow-based assay allows the assessment of primary hemostasis under physiological shear conditions, providing a more accurate and clinically relevant evaluation of platelet and VWF function. Such a study is possible using the Total Thrombus Formation Analysis System (T-TAS 01), initially designed to monitor the efficacy of antithrombotic agents, which allows analysis of hemostatic capacity in whole blood under a shear rate of 1500 s^-1^, mimicking blood flow in small arteries [18]. Under these conditions, platelet adhesion in human is highly dependent on VWF. A major advantage lies in the fact that this system can be used in thrombocytopenic conditions [19]. To our knowledge, this type of perfusion system has never been used to explore primary hemostasis in patients with cirrhosis.

Evaluating primary hemostasis under such physiological conditions would help to improve patients’ with cirrhosis management during the perioperative period. In particular, the management of thrombocytopenia and the question of prophylactic platelet transfusion often arise before an invasive procedure. Although the threshold of 30-50 G/L is based on a low level of evidence, platelet transfusion often shows limited efficacy in cirrhotic patients and may be associated with adverse effects [[20], [21], [22]]. Therefore, in the absence of strong data supporting its benefit, preventive platelet transfusion should be considered with caution and discussed on a case-by-case basis. A possible alternative to platelets transfusion could be synthetic platelet-mimicking nanoparticles (PMNPs). PMNPs are intravenous agents designed to mimic platelet adhesion and aggregation, with potential prophylactic or therapeutic applications in thrombocytopenic or bleeding patients. Current evidence is mainly preclinical, with no established thresholds or contraindications. As complementary strategies, thrombopoietin receptor agonists (e.g., avatrombopag and lusutrombopag) may increase platelet counts before elective procedures but require several days to act and carry a risk of thrombotic complications. PMNPs are made of a liposomal particle surface decorated with 3 different peptides: collagen binding peptide, which binds to fibrillar collagen exposed at the sub-endothelium, fibrinogen mimetic peptide, which can bind to the active form of platelet integrin αIIbβ3, and VWF binding peptide, which is derived from the C2 domain of factor VIII and can bind to the D'-D3 domain of VWF [[23], [24], [25]]. These PMNPs collaborate with activated fully functional and viable platelets to improve hemostasis *in vitro* and *in vivo* [26].

The aims of this study are (1) to evaluate alterations in perfusion parameters measured with the T-TAS 01 system in patients with cirrhosis compared with control patients and (2) to assess whether PMNPs can modulate or correct these alterations.

## 2 Patients and Methods

### 2.1 Patient selection

This observational study was conducted over an 8-months period (April 2024 to January 2025) at the Hepato-Biliary Centre of Paul Brousse Hospital (AP-HP, Université Paris-Saclay). The protocol was approved by the institutional ethics committee (Comité de Protection des Personnes Sud-Méditerranée III, 2023-A00064-41, April 20, 2023), and informed consent was obtained from all participants in accordance with the Declaration of Helsinki.

Two groups of adult patients were enrolled. The first group consisted of 30 patients with cirrhosis followed at Paul Brousse Hospital. The second group comprised 30 control patients without known cirrhosis, recruited from those undergoing nononcologic digestive surgery or upper/lower gastrointestinal endoscopy at the same institution.

Exclusion criteria include patients with hemostatic disorders unrelated to cirrhosis, patients receiving chronic antiplatelet or anticoagulant therapy, recent use of nonsteroidal anti-inflammatory drugs (within 5 days), genetically confirmed platelet function disorders, patients under estrogen therapy, active cancer or cancer treatment within the previous 6 months, current immunosuppressive or immunomodulatory therapy, and for the control group, any clinical or laboratory evidence suggestive of cirrhosis.

### 2.2 Routine biological tests

Blood cell count was performed on EDTA samples using a XN-1500 analyzer (Sysmex). Citrated plasma samples (sodium citrate 0.109 M [3.2%]) were prepared by double centrifugation at 2500 g for 15 minutes and used immediately or stored at –80 °C. Routine tests (prothrombin time [PT], activated partial thromboplastin time [aPTT], fibrinogen, and factor [F] II, FV, and FVIII) were performed using a STA-R Max analyser (Stago BioCare) using routine reagents from Stago (STA-NeoPTimal, STA-PTT, STA-Immunodeficient plasma) and Dade Thrombin Reagent for determining fibrinogen with Clauss method (Siemens).

Bilirubin and albumin concentrations were determined using a blood sample in a lithium-heparin tube. Total bilirubin concentration was determined using a photometric technique with routine reagents from Abbott. Total albumin concentration was determined using an immuno-turbidimetric technique, with routine reagents from DiAgam. Both techniques were performed on an Alinity analyzer (Abbott).

PT, bilirubin, and albumin are part of the Child-Pugh score (with ascites and encephalopathy), initially designed to predict outcomes in patients undergoing portal hypertensive surgery since 1973 and adopted to predict outcomes in patients undergoing transjugular intrahepatic portosystemic shunt creation since 1989 [27,28]. Each parameter is scored from 1 to 3, and the total score classifies patients into 3 classes: Class A (5-6 points): well-compensated disease (survival rate at 1 year 100%); Class B (7-9 points): significant functional compromise (survival rate at 1 year 80%); Class C (10-15 points): decompensated disease with poor prognosis (survival rate at 1 year 45%). This scoring system provides a standardized way to stratify patients by disease severity and helps guide clinical management and prognosis assessment.

### 2.3 VWF antigen, activity, propeptide, and multimer analysis

VWF antigen (VWF:Ag) dosage was performed with the STA Liatest VWF:Ag (Stago) and VWF activity (VWF:Act) was determined with the VWF:GPIbM Innovance kit (Siemens). Both techniques were performed on STA-R Max analyser (Stago).

The concentration of plasmatic VWF propeptide (VWF:pp) was determined with a commercial enzyme-linked immunoabsorbent assay (ELISA) kit using anti-human VWF:pp monoclonal antibodies (Senova).

The VWF multimer profiling was performed essentially as previously described, using 1.5% or 2% agarose gels [29]. VWF was detected with an in-house alkaline phosphatase-conjugated polyclonal anti-VWF and colorimetric alkaline phosphatase-substrate kit (Bio-Rad Laboratories). Membranes were imaged with a G:BOX Chemi XT16 Image Systems (Syngene). Multimer profiles were analyzed using the Gel Analyzer tool of ImageJ (version 1.53).

### 2.4 ADAMTS13 activity

ADAMTS13 activity (ADAMTS13:Act) was measured using our in-house fluorescence resonance energy transfer adapted from Kokame et al. [30], employing a commercial recombinant FRETS-VWF73 peptide (Peptide Institute) and standardized against the World Health Organization International Standard [31]. Briefly, ADAMTS13:Act was performed using a citrated plasma sample diluted in Bis-tris 5 mM (pH 6), CaCl_2_ (25 mM), Tween-20 (0.005%), and 0.22 mM Pefabloc. Proteolytic cleavage of the FRETS-VWF73 substrate by ADAMTS13 disrupts the intramolecular fluorescence quenching inherent to the intact peptide. Incubation of this substrate with a plasma sample from controls or patients with cirrhosis induces a time-dependent increase in fluorescence intensity, allowing quantitative assessment of ADAMTS13 enzymatic activity. Substrate conversion was monitored over 60 minutes. The normal range for ADAMTS13:Act is between 50 and 100 IU/dL (%). The upper limit of quantification for ADAMTS13:Act was 100%.

### 2.5 Microfluidic analysis and platelet mimicking nanoparticles

Thrombus formation under flow conditions was assessed using the Total Thrombus-formation Analysis System, with type I collagen-coated chip (PL chip) (T-TAS 01, Fujimori Kogyo) [32]. Whole blood anticoagulated with benzylsulfonyl-D-Arg-Pro-amidinobenzylamide (BAPA) was perfused at 37 °C through the microcapillary channel of the chip using a pneumatic pump. As the clot formed, the pressure in the perfusion chamber increased until total occlusion occurred. The parameters measured were: (i) the time required to achieve a pressure within the perfusion chamber equal to 10 kPa above the baseline pressure (occlusion start time, [OST]), ii) the time required to reach a pressure of 60 kPa above the base pressure in the infusion chamber (occlusion time, OT), and (iii) the area under the curve (AUC) at 10 minutes (area under the flow pressure curve, reflects the overall thrombus formation capacity over 10 minutes of perfusion). Each perfusion was performed in duplicate. For cirrhotic patients, perfusions were performed before and after the *in vitro* addition of PMNPs, at the ratio of 10, 25, or 50 particles per platelet. For the current studies, only first-generation PMNPs were used (liposomal particle surface decorated with 3 different peptides: collagen binding peptide, which binds to fibrillar collagen exposed at the sub-endothelium, fibrinogen mimetic peptide, which can bind to the active form of platelet integrin αIIbβ3, and VWF binding peptide, which is derived from the C2 domain of factor VIII and can bind to the D'-D3 domain of VWF), a design that has been previously shown to be efficient in basic thrombocytopenic conditions [33,34].

For a small subset of patients, perfusion experiments were also conducted using a parallel-plate flow chamber (Maastricht flow chamber). Briefly, benzylsulfonyl-D-Arg-Pro-amidinobenzylamide-anticoagulated blood was labeled with rhodamine 6G (10 μg/mL) for 5 minutes at room temperature and then perfused over a fibrillar type 1 collagen matrix (50 μg/mL; Chrono-log) for 2 minutes under arterial shear conditions (1500 s^-1^). This was followed by a 2-minute wash with Tyrode’s buffer (137 mM NaCl, 2 mM KCl, 0.3 mM NaH_2_PO_4_, 1 mM MgCl_2_, 5.5 mM glucose, 5 mM HEPES, and 12 mM NaHCO_3_; pH 7.3). Real-time thrombus formation was monitored using an inverted epifluorescence microscope (Nikon Eclipse TE2000-U, Nikon Instruments), coupled with Metamorph 7 software (Universal Imaging Corporation). Thrombus formation was quantified by measuring the mean percentage of the total surface area covered by thrombi [35].

### 2.6 Statistical analysis

Quantitative variables are expressed as median with interquartile range (25th-75th percentile), and qualitative variables as counts and percentages. Group comparisons were made using the Mann-Whitney or Kruskal-Wallis tests for nonparametric data. Paired tests were used to analyze changes before and after nanoparticle addition. Spearman correlation coefficients between biological parameters were determined, and a principal component analysis (PCA) was performed, followed by a hierarchical ascending classification (HAC). The cophenetic correlation coefficient of the HAC was calculated. This coefficient measures how faithfully a dendrogram preserves the dissimilarities among observations. A cophenetic correlation coefficient with a magnitude close to 1 indicates a high-quality solution.

The best equation reflecting the relationship between platelet count and T-TAS 01 AUC was searched. To decipher the determinants of the AUC, a multiple linear regression by stepwise model was conducted. The probability for entry was set at 0.05 and the probability of removal at 0.1. Missing data were estimated using the nearest neighbour technique.

A *P*-value < .05 was considered statistically significant, with Bonferroni correction applied in case of multiple comparisons. All statistical analyses were performed with the XLSTAT 2025 package (Addinsoft).

## 3 Results

From April 2024 to January 2025, 30 controls and 30 cirrhotic patients have been included. Among patients with cirrhosis 10 had Child-Pugh A cirrhosis, 10 Child-Pugh B, and 10 Child-Pugh C. Causes of cirrhosis were alcohol alone (*n* = 13), alcohol plus metabolic dysfunction-associated steatotic liver disease (*n* = 6), alcohol plus hepatitis virus B or C (*n* = 2), metabolic dysfunction-associated steatotic liver disease alone (*n* = 1), hepatitis virus B or C (*n* = 2), biliary cause (*n* = 2), Wilson disease (*n* = 2), Wilson disease plus alcohol (*n* = 1) and autoimmune cirrhosis (*n* = 1). Patients’ characteristics are detailed in Table. Patients with cirrhosis exhibited significant hemostatic alterations, more severe in Child-Pugh B and C patients than in Child-Pugh A patients. VWF:pp was significantly increased in patients with cirrhosis, with a higher VWF:pp/VWF:Ag ratio in the more severe patients. ADAMTS13:Act was lower in patients with cirrhosis and reduced below 50% in 10 patients, with the VWF:Ag/ADAMTS13:Act ratio increasing with the severity of cirrhosis. VWF multimer gel analysis did not exhibit a significant increase of the high-molecular-weight VWF multimers (data not shown).

**Table:** Patients’ characteristics

	Control	Cirrhotic	Child A	Child B	Child C
	(*n* = 30)	(*n* = 30)	(*n* = 10)	(*n* = 10)	(*n* = 10)
Sex (male)	15 (50.0)	23 (76.7)∗	8 (80)	8 (80)	7 (70)
Age (y)	56 (46-64)	58 (49-64)	56 (35-65)	56 (51-62)	62 (49-65)
BMI (kg/m^2^)	25.1 (22.0-27.9)	25.6 (21.5-27.8)	27.5 (24.7-27.8)	24.4 (21.1-28.2)	22.5 (21.4-25.7)
Leukocytes (G/L)	5.90 (5.28-6.51)	4.73 (3.52-7.05)	4.18 (3.86-5.76)	4.14 (2.37-6.92)	6.34 (4.15-7.60)
Hemoglobin (g/dL)	14.1 (12.9-14.8)	11.9 (10.3-13.6) ∗∗∗	14.1 (12.3-14.9)	11.8 (9.9-12.4) ∗∗	11.3 (10.1-12.2) ∗∗
Hematocrit (%)	40.9 (38.2-43.5)	35.9 (31.0-40.4) ∗∗∗	41.5 (37.8-43.3)	34.6 (29.3-35.6) ∗∗ ∗	34.4 (31.0-36.7) ∗∗ ∗
Platelets (G/L)	218 (201-278)	97 (75-130) ∗∗∗∗	116 (84-138) ∗∗	96 (86-106) ∗∗∗	90 (65-126) ∗∗∗
Prothrombin time (%)	92 (84-99)	52 (45-67) ∗∗∗∗	77 (70-80)	52 (48-59) ∗∗∗	45 (33-48) ∗∗∗ ∗
Factor II (%)	98 (93-101)	56 (43-70) ∗∗∗∗	82 (72-85)	53 (44-62) ∗∗∗	39 (32-42) ∗∗∗ ∗
Factor V (%)	105 (94-124)	60 (47-78) ∗∗∗∗	78 (66-109)	57 (48-72) ∗∗∗	43 (32-58) ∗∗∗
aPTT ratio	1.02 (0.96-1.09)	1.24 (1.07-1.36) ∗∗∗	1.01 (0.91-1.06)	1.29 (1.20-1.41) ∗∗∗ ∗∗	1.35 (1.25-1.41) ∗∗∗ ∗∗∗
Fibrinogen (g/L)	3.39 (3.04-3.82)	2.73 (2.27-3.40) ∗∗	3.34 (2.78-3.52)	3.01 (2.25-3.34)	2.30 (1.79-2.63) ∗∗ ∗
Factor VIII (%)	129 (105-168)	193 (131-240) ∗∗∗	181 (151-230)	169 (130-256)	218 (137-233) ∗
VWF:Act GPIbM (%)	124 (107-159)	334 (231-400) ∗∗∗∗	280 (163-258) ∗∗	349 (282-392) ∗∗∗	388 (251-400) ∗∗∗
VWF:Ag (%)	119 (100-141)	310 (245-347) ∗∗∗∗	276 (149-333) ∗∗	315 (252-350) ∗∗∗	319 (295-343) ∗∗∗
VWF:Ag/platelets ratio	0.53 (0.39-0.71)	3.00 (2.04-4.23) ∗∗∗∗	2.57 (1.41-3.44) ∗∗	3.20 (2.35-3.79) ∗∗∗	3.33 (2.46-4.96) ∗∗∗
VWF:propeptide (%)	173 (128-281)	1172 (743-1573) ∗∗∗∗	676 (205-955)	1281 (858-1578) ∗∗∗	1557 (1276-2441) ∗∗∗
VWF:pp/VWF:Ag ratio	1.42 (1.12-2.83)	4.02 (2.36-5.23) ∗∗∗	2.27 (1.35-3.45)	4.02 (2.93-5.87) ∗	4.89 (4.67-6.95) ∗∗∗ ∗
ADAMTS13:Act (%)	100 (93-100)	77 (43-100) ∗∗	82 (71-100)	51 (43-98)	56 (28-100)
VWF:Ag/ADAMTS13:Act ratio	1.22 (1.00-1.57)	4.07 (2.74-7.99) ∗∗∗∗	3.41 (1.89-4.25) ∗	5.20 (3.12-7.99) ∗∗∗	7.56 (2.97-12.52) ∗∗∗
Total bilirubin (μmol/L)	14 (9-18)	26 (17-57) ∗∗∗∗	18 (16-24)	21 (18-27)	62 (47-80) ∗∗∗ ∗
Albumin (g/L)	40.7 (39.4-45.4)	33.2 (28.2-37.9) ∗∗∗∗	39.0 (7.4-41.1)	28.9 (28.0-34.2) ∗∗∗ ∗	31.4 (28.5-33.3) ∗∗∗ ∗
Occlusion start time	02:21	05:08	04:22	05:09	06:56
(min:sec)	(02:10-02:32)	(02:53-09:28) ∗∗∗∗	(02:34-07:30) ∗	(02:50-07:35) ∗	(04:07-09:55) ∗∗∗
AUC	382 (348-391)	96 (17-282) ∗∗∗∗	152 (49-322) ∗∗	94 (49-249) ∗∗∗	60 (6-155) ∗∗∗
Occlusion time	05:00	10:00	10:00	10:00	10:00
(min:sec)	(04:38-05:50)	(07:13-10:00) ∗∗∗∗	(06:24-10:00) ∗∗	(08:16-10:00) ∗∗∗	(10:00-10:00) ∗∗∗

Continuous and categorical variables expressed respectively as median (IQR) and n (%). Act, activity; ADAMTS13, a disintegrin-like metalloproteinase with thrombospondin type-1 motifs 13; Ag, antigen; aPTT, activated partial thromboplastin time; AUC, area under the curve; BMI, body mass index; FVIII, factor VIII; pp, propeptide; VWF, von Willebrand factor.

Comparison between cirrhotic and control; Mann-Whitney test or Χ^2^ test:

∗*P* < 0.05.

∗∗*P* < 0.01.

∗∗∗*P* < 0.001.

∗∗∗∗*P* < 0.0001.

Comparison between Child A, Child B, Child C, and control; Kruskal-Wallis test (Bonferroni corrected signification level = 0.0083). - Between Child A and control: ∗*P* < .008, ∗∗*P* < .001, ∗∗∗*P* < .0001 - Between Child B and control: ∗*P* < .008, ∗∗*P* < .001, ∗∗∗*P* < .0001

Between Child C and control: ∗*P* < .008, ∗∗*P* < .001, ∗∗∗*P* < .0001. - Between Child B and Child A: ∗*P* < .008, ∗∗*P* < .001, ∗∗∗*P* < .0001 - Between Child C and Child A: ∗*P* < .008, ∗∗*P* < .001, ∗∗∗*P* < .0001

**Figure 1:**
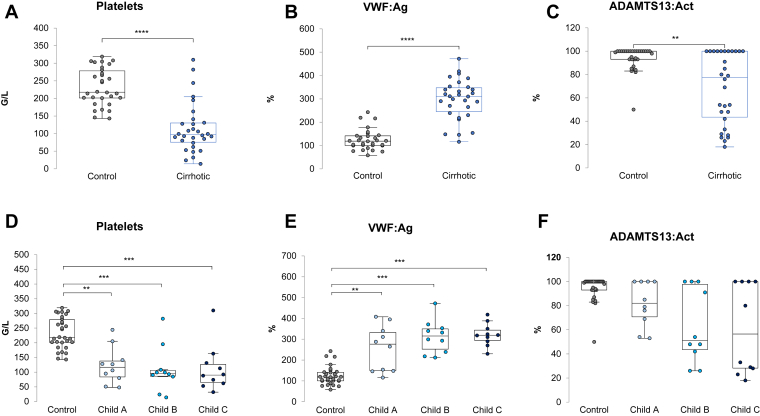
Platelet count, VWF:Ag and ADAMTS13 activity. (A), (B), and (C) Comparison of control (gray circles) and cirrhotic (blue circles) patients. Data were compared with Mann-Whitney test: ∗*P* < .05, ∗∗*P* < .01, ∗∗∗*P* < .001. (D), (E), and (F) Comparison between control patients (gray circles), Child A (light blue circles), Child B (turquoise blue circles), and Child C patients (dark blue circles). Data were compared with Kruskal-Wallis test with Bonferroni correction: ∗*P* < .008, ∗∗*P* < .001, ∗∗∗*P* < .0001. Act, activity; ADAMTS13, a disintegrin-like and metalloproteinase with thrombospondin type-1 motifs 13; Ag, antigen; VWF, von Willebrand factor.

Individual results of platelets count, VWF:Ag and ADAMTS13:Act are presented in Figure 1. Patients with cirrhosis Cirrhotic patients had significantly lower platelet counts, higher VWF:Ag, and lower ADAMTS13:Act than control patients. These differences were more marked with increasing severity of cirrhosis.

**Figure 2:**
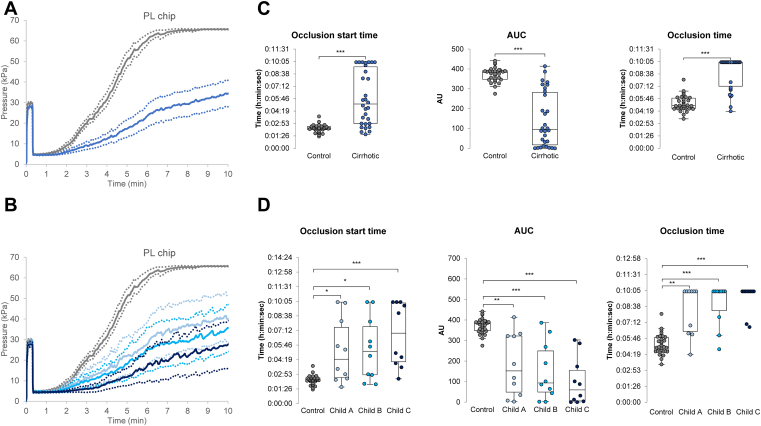
T-TAS 01 perfusions performed on PL chips. Pressure curves obtained (A) for control (gray, *n* = 30) and cirrhotic (blue, *n* = 30) patients or (B) for control (gray, *n* = 30), Child A (light blue, *n* = 10), Child B (turquoise blue, *n* = 10) and Child C (dark blue, *n* = 10) cirrhotic patients. Curves are represented as mean (solid line) and 95% CI (dotted lines). (C) Comparison of occlusion start time, AUC and occlusion time of control (gray circles) and cirrhotic (blue circles) patients. Note that the perfusions ran for a maximum of 10 minutes. Data were compared with Mann-Whitney test: ∗*P* < .05, ∗∗*P* < .01, ∗∗∗*P* < .001. (D) Comparison of occlusion start time, AUC and occlusion time between control patients (gray circles), Child A (light blue circles), Child B (turquoise blue circles), and Child C patients (dark blue circles). Data were compared with Kruskal-Wallis test with Bonferroni correction: ∗*P* < .008, ∗∗*P* < .001, ∗∗∗*P* < .0001. AU, arbitrary unit; AUC, area under the curve.

The results of the perfusions performed on T-TAS 01 with PL chips are presented in Figure 2. Patients with cirrhosis exhibited significantly longer OST and occlusion time (OT) and lower AUC at 10 minutes than the control. These differences increased with cirrhosis severity, while platelet count decreased and VWF:Ag increased.

**Figure 3:**
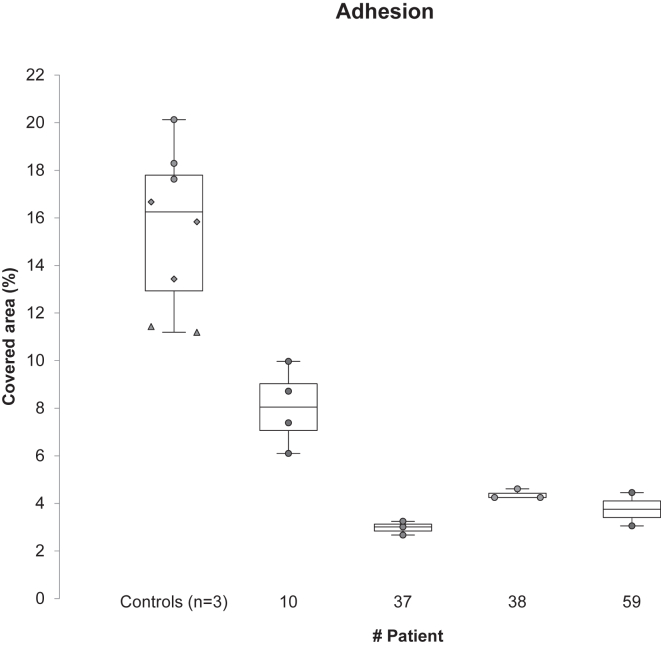
Perfusions performed on parallel-plate flow chamber of 3 control and 4 cirrhotic patients (*n* = 7). Percentage of covered area represents platelet adhesion. Each dot represents one perfusion. Each control patient is represented by circles, diamonds or triangles.

To corroborate these results, blood from 3 controls and 4 patients with cirrhosis randomly selected were also explored in parallel-plate flow chamber (*n* = 7). Figure 3 shows the percentage of surface area covered by thrombi in the Maastricht chamber, reflecting platelet adhesion. Patients with cirrhosis exhibited lower platelet adhesion than control. In patients #37, 38, and 59, the high VWF:Ag did not compensate for thrombocytopenia. Patient #10 who had the highest VWF:Ag still showed a slightly higher platelet adhesion, although not normalized. Thrombi sizes were also measured, using mean fluorescence intensity, and the results were comparable to those of platelets adhesion (data not shown).

**Figure 4:**
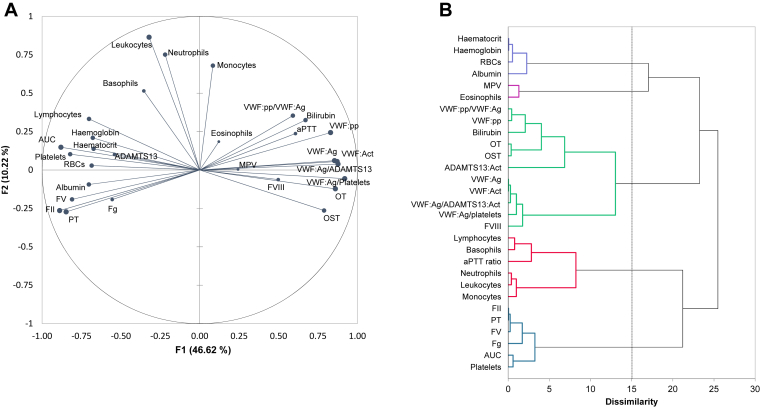
(A) Principal component analysis including all biological parameters and T-TAS 01 results from all control and cirrhotic patients. (B) Hierarchical ascending classification. Act, activity; ADAMTS13, a disintegrin-like and metalloproteinase with thrombospondin type-1 motifs 13; Ag, antigen; aPTT, activated partial thromboplastin time; AUC, area under the curve; FII, factor II; FV, factor V; FVIII, factor VIII; Fg, fibrinogen; MPV, mean platelet volume; pp, propeptide; OST, occlusion start time; OT, occlusion time; PT, prothrombin time ratio; RBCs, red blood cells; VWF, von Willebrand factor.

To decipher the determinants of the T-TAS 01 results, a PCA was conducted, followed by a HAC. On the PCA (Figure 4A), the x- and y-axis (F1 and F2) represented 57% of the total variability of the scatter plot. The larger the dot, the higher the squared cosines, reflecting the representation quality of a variable on the PCA axis. Noteworthy, dots are not interpretable if they are too close to the center. The main component (x-axis, 47% of variability) strongly associated in a common cluster highly correlated parameters: coagulation factors, platelets, VWF:Ag and VWF:Act, ADAMTS13:Act, VWF:pp and T-TAS 01 occlusion start time, occlusion time, and AUC. Looking at secondary hemostasis, PT, FII, FV, and Fg were positively correlated with each other (Spearman correlation coefficients between 0,64 and 0.91, *P* < .05), and negatively correlated with aPTT (Spearman correlation coefficients between −0.71 and −0.52, *P* < .05). The second component (y-axis, 10% of variability) concerned only leukocytes populations. Figure 4B shows the dendrogram of the HAC. Platelets and AUC clustered together, whereas OST and OT clustered with VWF and its regulator ADAMTS13. Secondary hemostasis parameters (PT, FII, FV, and Fg) clustered together, whereas FVIII clustered with VWF. The cophenetic correlation coefficient of the HAC was 0.68.

**Figure 5:**
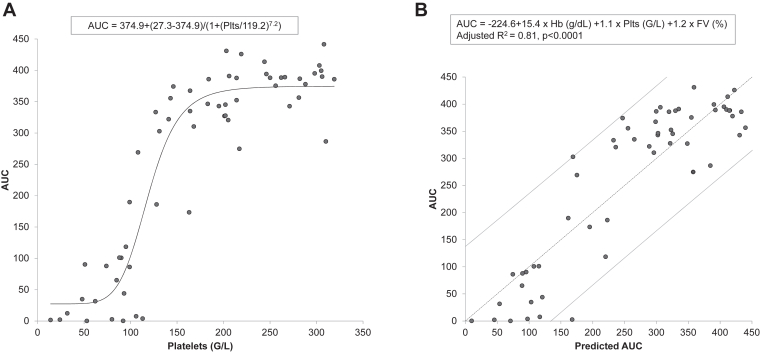
(A) Nonlinear regression between platelets and T-TAS 01 AUC. (B) Multiple linear regression by stepwise model. The dark dotted line represents the model and the gray line the 95% CI. Hb, hemoglobin; FV, factor V; Plts, platelets.

The relationship between platelet count and AUC was best represented by a 4 parameters logistic equation (Figure 5A). To decipher the determinants of the AUC, a multiple linear regression by stepwise model was conducted (Figure 5B). Eighty-one % of the AUC variability was explained by hemoglobin level, platelet count, and FV activity (*P* < .0001), platelet count being the most influential variable, followed by hemoglobin level and FV activity. The same analysis conducted for OST showed that 65% of the variability was explained by hemoglobin level, platelet count (the most influent variable), and bilirubin (*P* < .0001). Looking at OT, 84% of the variability were explained by neutrophils, platelet count (the most influent variable), FII activity, VWF:Ag, and ADAMTS13:Act (*P* < .0001).

**Figure 6:**
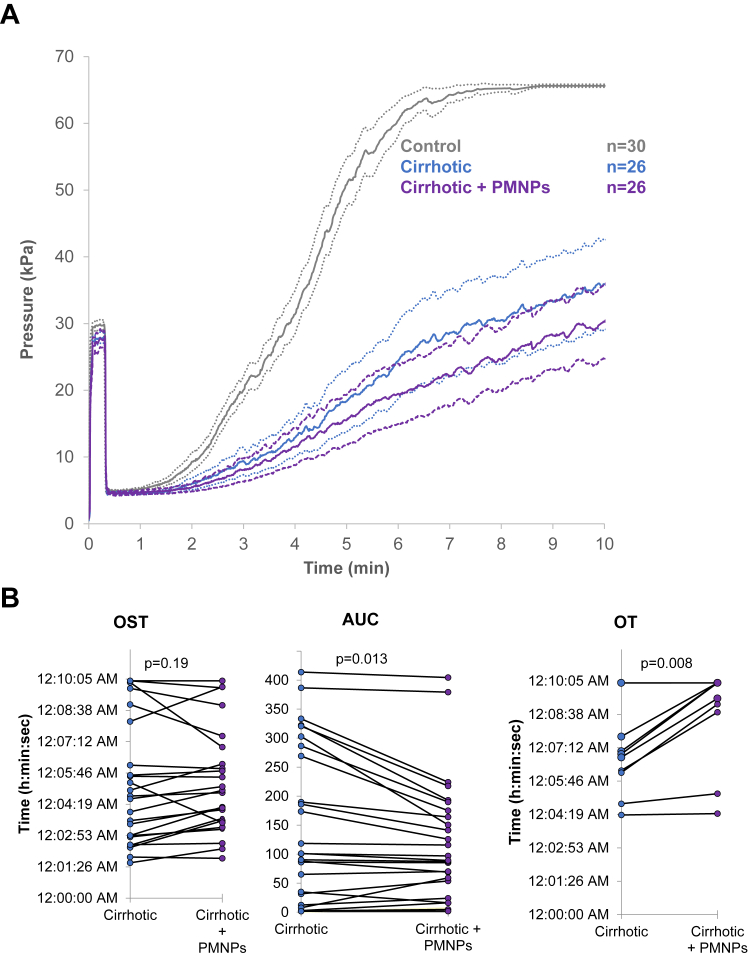
(A) T-TAS 01 perfusions performed on PL chips. Pressure curves obtained for control (gray, *n* = 30), cirrhotic (blue, *n* = 26) and cirrhotic patients plus platelets mimicking nanoparticles at the ratio of 50 PMNPs per platelet (purple, *n* = 26). Curves are represented as mean (solid line) and 95% CI (dotted lines). (B) Comparison of occlusion start time (OST), AUC and occlusion time (OT) of cirrhotic (blue circles) and cirrhotic + 50 PMNPs per platelet (purple circles) patients. Note that the perfusions ran for a maximum of 10 minutes. Data were compared with Wilcoxon rank-sum test for paired data. Act, activity; ADAMTS13, a disintegrin-like metalloproteinase with thrombospondin type-1 motifs 13; Ag, antigen; aPTT, activated partial thromboplastin time; AUC, area under the curve; BMI, body mass index; FVIII, factor VIII; Fg, fibrinogen; HAC, hierarchical ascendant classification; MPV, mean platelet volume; OST, occlusion start time; OT, occlusion time; PCA, principal component analysis; pp, propeptide; RBCs, red blood cells; VWF, von Willebrand factor.

For 26 patients with cirrhosis, T-TAS 01 perfusions were also performed after *in vitro* addition of 50 PMNPs per platelet. PMNPs had no effect on OST and decreased AUC and OT (Figure 6). Moreover, the slope of the cirrhotic + PMNPs curve was less steep than that of cirrhotic alone. The effect of PMNPs was highly variable, as shown in Figure 6B. PMNPs had a positive effect on AUC in 8/26 patients, whose platelet count was significantly lower (71 [30-87] vs 117 [91-157], *P* = .003). Decreasing the PMNPs/platelet ratio (10 or 25 PMNPs per platelet) did not improve the perfusion parameters, and increasing the ratio (500 PMNPs per platelet) seemed to worsen them (data not shown).

In control patients, the addition of PMNPs was tested in the Maastricht flow chamber, and did not show any effect (data not shown).

## 4 Discussion

The main objective of our study was to investigate whether a microfluidic-based hemostasis assessment technology like T-TAS 01 could be utilized to assess hemostatic capability of patients with cirrhosis. The second objective was to test whether the addition of first-generation PMNPs could serve as a potential alternative to platelet transfusion in improving hemostatic parameters.

Exploration of whole blood from patients with cirrhosis was performed using the T-TAS 01 PL chips, coated with collagen. The decision to use PL-chips was motivated by their specific design for the evaluation of primary hemostasis, in both normal platelet count and thrombocytopenic conditions. Other available chips (AR and HD) are coated with collagen and tissue factor, thereby reflecting secondary hemostatic mechanisms. As our objective was to focus exclusively on primary hemostasis, the AR-and-HD chip was not included in this study, but its comparison with PL-chips could provide complementary insights and will be investigated in future work [36].

Our results showed significantly altered primary hemostasis in patients with cirrhosis compared with control. A striking observation is that the significant elevation of VWF:Ag and VWF:Act did not compensate for the thrombocytopenia. This was confirmed by the perfusions performed applying the Maastricht flow chamber, used as a complementary mechanistic assay. Both models used chips coated with collagen I, and perfusions were performed under arterial shear conditions (1500 s^-1^). At first sight, these results may seem at odds with those of Lisman et al. [11], who found that, in adult patients with cirrhosis, the elevated VWF levels compensated for thrombocytopenia, resulting in subnormal thrombus formation [11]. One major explanation may lie in the type of perfusions used. Whereas all our perfusions were performed using patients’ native whole blood, Lisman et al. [11] used a reconstituted blood model, with a hematocrit of 40% and a fixed platelet count. Such an approach has the advantage of investigating the different parameters (plasma vs blood cells) individually but may mask a potential global effect. Indeed, and in contrast to Lisman’s study, Van Dievoet et al. [37] found a decreased platelet adhesion and aggregation in whole blood perfusions of paediatric cirrhotic patients compared to controls, using a Maastricht flow chamber at a shear rate of 1000 s^-1^.

Our study confirms and extends this last report to the adult context with the use of the T-TAS 01, an automated user-friendly system designed to be used in the routine laboratory or even at the patient’s bedside. Our approach using native whole blood also allowed us to explore correlations between hemostatic outputs and biological measurements. We found that the AUC measured in the T-TAS 01 perfusions highly correlated with platelet count. This result is in accordance with previous reports using the T-TAS 01 in healthy individuals [18]. Among the parameters highlighted by the PCA and the HAC, VWF:Ag, and VWF:pp were positively correlated with OST and OT and negatively with AUC. This seems counterintuitive, as one may expect a positive effect of VWF on thrombus formation. A potential explanation may lie in the increased VWFpp/VWF:Ag ratio measured in patients with cirrhosis compared with controls. Indeed, although both VWF:Ag and VWF:pp were increased in our patients, as previously described [11,38]. Furthermore, VWFpp was elevated and it has been described that increased VWF:pp levels may exert a negative regulation on VWF function in particular on VWF-dependent platelet adhesion [39,40]. In our study, the VWF:Ag/ADAMTS13:Act ratio increased with the severity of cirrhosis, consistent with previous reports [41].

Another important parameter affecting results of the T-TAS 01 perfusions (OST and AUC) are red blood cells (RBCs) and its corollaries hemoglobin and hematocrit. RBCs are the major cellular component of blood and are known to affect platelet adhesion notably by promoting platelet margination near the vessel-wall [42]. RBCs also interact directly with and activate platelets during high shear conditions [[42], [43], [44], [45]]. Yaoi et al. [46] have previously demonstrated the role of hematocrit in OST and AUC of T-TAS 01 perfusions performed on PL chips at 1000 s^-1^ [46]. In our study, patients with cirrhosis had significantly lower RBCs than the control, and this may have contributed to the altered thrombus formation.

While platelet count provides a quantitative measure, T-TAS 01 offers a more comprehensive assessment of the thrombus formation process, integrating a global aspect of primary hemostasis under flow conditions with PL-chips. This functional evaluation can help identify patients with preserved hemostatic capacity despite thrombocytopenia. Therefore, T-TAS 01 may be particularly useful in clinical situations where platelet counts are low but T-TAS 01 parameters remain within normal or reassuring ranges, potentially helping to avoid unnecessary platelet transfusions before high-risk invasive procedures. In patients with cirrhosis, for example, T-TAS 01 could help identify those who maintain adequate hemostatic potential despite thrombocytopenia. This may be especially useful for preprocedural risk stratification before high-bleeding-risk interventions, guiding transfusion decisions, and monitoring hemostatic changes following therapeutic interventions. Prospective studies are required to establish whether T-TAS 01 can be used to tailor clinical management in this population.

Recent studies using whole-blood coagulation assays further support our observation of impaired thrombus formation in cirrhosis. Zanetto et al. [47] demonstrated a hypocoagulable profile in patients with decompensated cirrhosis when using whole-blood thrombin generation, in contrast to platelet-poor plasma assays. In another prospective cohort of 231 patients, whole-blood thrombin generation predicted procedure-related bleeding, whereas plasma-based assays did not [48]. These findings are consistent with our results obtained using T-TAS 01, which assesses thrombus formation in whole blood under flow condition. Together, these data suggest that global, flow-based whole-blood assays provide a more physiologically relevant assessment of the hemostatic alterations in cirrhosis and may help identify patients with preserved thrombus-forming capacity despite low platelet counts or abnormal conventional tests, thereby informing procedural planning and transfusion decisions.

The last part of our study consisted in evaluating the therapeutic potential of PMNPs. In the current study setting PMNPs were found to only partly improve T-TAS 01 parameters in a subset of patients with cirrhosis, interestingly those with a lower range of platelet counts. While such PMNPs have been previously shown to be effective in rescuing primary hemostasis in anti-GPIb-induced thrombocytopenic conditions in mice [33], the current study indicates that the interaction of PMNP with platelets of patients with cirrhosis may be more nuanced. Indeed, this first-generation PMNPs has been designed to rescue primary hemostatic function in a setting of straightforward thrombocytopenia [33], but it may have limited capabilities if functional defects are superposed on count defects. It is also important to mention that PMNPs have been designed as a therapeutic approach in traumatic injury with massive blood loss where there is platelet number depletion but there may not be significant compromise in residual platelet function [[49], [50], [51]]. Their efficacy in other pathological conditions has only been shown in von Willebrand disease murine models [34] and efficacy in models of defective platelet function are yet to be investigated. Moreover, new generations of PMNPs, such as PMNPs loaded on the surface with procoagulant lipids or loaded in the core with pro-hemostatic cargo have been designed to achieve adjunctive hemostatic capabilities beyond the original PMNPs tested in the present study [52,53]. Effect of such next-generation PMNPs in patient with cirrhosis blood and other human patient blood with potential platelet functional defects remains to be tested.

This study has several limitations. First, it is a monocentric study. Second, various etiologies of cirrhosis were present among patients, and results must be confirmed in a larger cohort. Moreover, patients with cirrhosis were in a stable condition, and hemostasis may be more impaired in decompensated or acute-on-chronic liver failure patients. Third, we did not explore the possibility of platelet functional defects in our current cohort of patients with cirrhosis. Fourth, and according to the manufacturer's documentation, PL chips have not been validated for samples with platelet counts below 114 × 10^3^/μL and/or hematocrit values below 25%. As thrombocytopenia is common in cirrhotic patients, this technical limitation should be considered when interpreting T-TAS 01 results. Fifth, only one type of platelet-mimicking nanoparticles was tested.

## 5 Conclusion

In summary, T-TAS 01 perfusions with PL chips provided insights into primary hemostasis alterations in patients with cirrhosis, positioning this study as a physiological and methodological proof-of-concept. Elevated VWF levels did not appear to fully compensate for thrombocytopenia and may further alter platelet thrombus formation. First-generation PMNPs showed only partial effects in this perfusion model. While these findings suggest that T-TAS 01 could have potential applications in clinical settings, further studies are required to confirm these observations, like investigating the respective roles of VWF:Ag and VWF:pp, exploring platelet functions more comprehensively, and evaluating new generations of PMNPs.
